# Enhancement in Crystallizability of Poly(*L*-Lactide) Using Stereocomplex-Polylactide Powder as a Nucleating Agent

**DOI:** 10.3390/polym14194092

**Published:** 2022-09-29

**Authors:** Yodthong Baimark, Prasong Srihanam, Yaowalak Srisuwan, Theeraphol Phromsopha

**Affiliations:** Biodegradable Polymers Research Unit, Department of Chemistry and Centre of Excellence for Innovation in Chemistry, Faculty of Science, Mahasarakham University, Mahasarakham 44150, Thailand

**Keywords:** poly(lactic acid), stereocomplex, biocomposites, nucleating agent, crystallization

## Abstract

High-molecular-weight poly(*L*-lactide) (HMW-PLLA) is a promising candidate for use as a bioplastic because of its biodegradability and compostability. However, the applications of HMW-PLLA have been limited due to its poor crystallizability. In this work, stereocomplex polylactide (scPLA) powder was prepared by precipitation of a low-molecular-weight poly(*L*-lactide)/poly(*D*-lactide) (LMW-PLLA/LMW-PDLA) blend solution and investigated for use as a fully-biodegradable nucleating agent for HMW-PLLA compared to LMW-PLLA powder. The obtained LMW-PLLA and scPLA powders with a nearly spherical shape showed complete homo- and stereocomplex crystallites, respectively. HMW-PLLA/LMW-PLLA powder and HMW-PLLA/scPLA powder blends were prepared by melt blending. The LMW-PLLA powder was homogeneously melted in the HMW-PLLA matrices, whereas the scPLA powder had good phase compatibility and was well-dispersed in the HMW-PLLA matrices, as detected by scanning electron microscopy (SEM). It was shown that the enthalpies of crystallization (Δ*H_c_*) upon cooling scans for HMW-PLLA largely increased and the half crystallization time (*t_1/2_*) dramatically decreased as the scPLA powder content increased; however, the LMW-PLLA powder did not exhibit the same behavior, as determined by differential scanning calorimetry (DSC). The crystallinity content of the HMW-PLLA/scPLA powder blends significantly increased as the scPLA powder content increased, as determined by DSC and X-ray diffractometry (XRD). In conclusion, the fully biodegradable scPLA powder showed good potential for use as an effective nucleating agent to improve the crystallization properties of the HMW-PLLA bioplastic.

## 1. Introduction

Poly(*L*-lactic acid) or poly(*L*-lactide) (PLLA) has been widely investigated for use in biomedical, controlled-release drug delivery, tissue engineering, and packaging applications [1,2,3,4]. This is because of its biodegradability, biocompatibility, and compostability as well as good processability and mechanical properties [5,6,7]. However, the poor crystallizability of PLLA is an important limiting factor restricting its use in many applications [8]. Processed PLLAs resulted in low-crystallinity-content products that have poor heat-resistance [9]. Moreover, the biodegradability and barrier properties of PLLAs strongly depended on PLLA crystallinity [8]. Various methods have been investigated to enhance the crystallization ability of PLLA, such as by nucleating-agent addition [8,9] and plasticizer blending [8], flow-induced crystallization [10], and stereocomplexation [11,12,13]. The addition of organic and inorganic nucleating agents has been extensively and commonly used for the promotion of PLLA crystallinity by reducing the nucleating barrier and increasing nucleating sites [8,14,15]. However, fully biodegradable and biocompatible nucleating agents still remain to be investigated.

Stereocomplex polylactides (scPLAs) are polymer blends between PLLA and poly(*D*-lactide) (PDLA) [11,12,13]. The melting temperatures of scPLAs (>200 °C) are higher than those of PLLA (150–170 °C) due to the strong van der Waals forces in the stereocomplex crystallites. Stereocomplex crystallites in PLLA-based polymers containing a small amount of PDLA act as nucleating sites for PLLA homo-crystallization [16,17,18]. However, stereocomplex crystallites may exhibit the tethering effect that interferes with the PLLA homo-crystallization growth by reducing PLLA chain-mobility [8,17]. The extent of PLLA homo-crystallization, then, does not increase with the content of stereocomplex-crystallite nucleating sites.

The preparation of scPLA powder resulting in complete stereocomplexation has rarely been published [19,20,21]. The scPLA powders prepared by supercritical fluid technology have been utilized as a nucleating agent for PLLA [20,21]. The use of high-cost PDLA was reduced by up to a half when scPLA powder was used instead of the PDLA homopolymer. However, the effectiveness of the scPLA powder prepared by a simple precipitation method on the crystallization properties of PLLA has not yet been investigated in detail.

In this paper, scPLA powder was prepared by the precipitation of a low-molecular-weight PLLA/low-molecular-weight PDLA (LMW-PLLA/LMW-PDLA) blend solution before melt blending with high-molecular-weight PLLA (HMW-PLLA). We hypothesize that the scPLA powder dispersed in the HMW-PLLA matrix could act as heterogeneous phases to enhance the nucleation effect for HMW-PLLA. The crystallization properties of HMW-PLLA were determined from non-isothermal and isothermal differential scanning calorimetry (DSC) analyses. The crystallinity content of HMW-PLLA-based films were also investigated by X-ray diffractometry (XRD) in order to better understand the nucleating efficiency of the scPLA powder. The LMW-PLLA powder was also prepared and was melt blended with HMW-PLLA for comparison.

## 2. Materials and Methods

### 2.1. Materials

LMW-PLLA and LMW-PDLA were synthesized by a ring-opening polymerization of *L*-lactide and *D*-lactide monomers, respectively, at 165 °C for 2.5 h under nitrogen atmosphere using stannous octoate (95%, Sigma, St. Louis, MO, USA) and 1-dodecanol (98%, Fluka, Buchs, Switzerland) as the initiating system [22]. Characteristics of the obtained LMW-PLLA and LMW-PDLA are summarized in Table 1. PLLA3251D (NatureWorks LLC, Plymouth, MN, USA) with a number-average molecular weight (*M_n_*) of 93,200 g/mol and a dispersity index (*Ɖ*) of 1.66 [23] was used as an HMW-PLLA. Dichloromethane (99.8%, RCI Labscan, Bangkok, Thailand) and ethanol (99.9%, RCI Labscan, Bangkok, Thailand) in analytical grade were chosen as a solvent and a non-solvent, respectively, for preparation of PLLA and scPLA powders.

### 2.2. Preparation of LMW-PLLA and scPLA Powders

A precipitation method was used to prepare scPLA powder. Briefly, 10 mL each of 20% w/v LMW-PLLA and 20% w/v LMW-PDLA solutions in dichloromethane were mixed together under magnetic stirring for 5 min before pouring into 200 mL of ethanol with continuous magnetic stirring for 2 h. The precipitated scPLA powder was collected by centrifugation at a relative centrifugal force (RCF) of 4082 for 60 min before drying in a vacuum oven at room temperature for 24 h. LMW-PLLA/LMW-PDLA ratio was 50/50% wt. LMW-PLLA powder was also prepared from LMW-PLLA solution by the same method for comparison.

### 2.3. Characterization of LMW-PLLA and scPLA Powders

Particle morphology of the powders was determined using a scanning electron microscope (SEM, JSM-6460LV, JEOL, Tokyo, Japan) at 20 kV. The powder samples were sputter coated with gold to avoid charging before scanning.

Particle sizes of the powders were investigated using a Zetasizer dynamic light- scattering analyzer (Zetasizer Nano ZS, Malvern Panalytical, Malvern, UK) at 25 °C. Powder samples were suspended in ethanol before measurement.

Melting temperature (*T_m_*) of the powder samples was determined using a differential scanning calorimeter (DSC, Pyris Diamond, PerkinElmer, Waltham, MA, USA). The sample was heated from 0 to 250 °C at a rate of 10 °C/min under a nitrogen gas flow. The degree of homo-crystallinity from DSC (DSC-*X_c,hc_*) and degree of stereocomplex crystallinity from DSC (DSC-*X_c,sc_*) of the powders were calculated using the Equations (1) and (2), respectively.

DSC-*X_c,hc_* (%) = (Δ*H_m,hc_*/93.6) × 100 (1)

DSC-*X_c,sc_* (%) = (Δ*H_m,sc_*/142) × 100 (2)

where Δ*H_m,hc_* and Δ*H_m,sc_* are enthalpies of melting for homo- and stereocomplex crystallites, respectively. In addition, 93.6 and 142 J/g are the Δ*H_m_* values for 100%*X*_c_ PLLA and scPLA, respectively [24,25,26].

Crystalline structures of the powders were measured using a wide-angle X-ray diffractometer (XRD, D8 Advance, Bruker Corporation, Karlsruhe, Germany) in the angle range of 2θ = 5°–30° equipped with a copper tube operating at 40 kV and 40 mA producing Cu − Kα radiation. Scan speed was 3°/min.

### 2.4. Preparation of PLLA/LMW-PLLA Powder and PLLA/scPLA Powder Blends

PLLA3251D and scPLA powders were dried at 50 °C in a vacuum oven overnight before melt blending using a batch mixer (HAAKE Polylab OS Rheomix, Thermo Scientific, Waltham, MA, USA) at 180 °C for 5 min. Rotor speed was 100 rpm. PLLA3251D-based blends with 2%, 4%, and 8% *w*/*w* scPLA powders were investigated. PLLA3251D/LMW-PLLA powder blends were also prepared by the same process for comparison.

Both the PLLA3251D/LMW-PLLA powder and PLLA3251D/scPLA powder blends were hot pressed to form film samples (100 mm × 100 mm) using a hot-press machine (Carver Auto CH, Wabash, IN, USA). The blends were dried at 50 °C in a vacuum oven overnight before hot pressing. The blends were preheated for 3 min at 180 °C before hot pressing for 1 min with 4 MPa force. The blend films were cooled to room temperature using a water-cooled press for 1 min with the same force. The blend films with 0.3–0.4 mm in thickness were kept in a desiccator for 24 h before characterization.

### 2.5. Characterization of PLLA/LMW-PLLA Powder and PLLA/scPLA Powder Blends

Phase morphology of the blend films was determined using a SEM (JSM-6460LV, JEOL, Tokyo, Japan). The blend films were cryogenically fractured after immersing in liquid nitrogen for 10 min and were sputter coated with gold before scanning at an acceleration voltage of 20 kV. 

The crystallization behaviors of the blends were examined on a DSC (Pyris Diamond, PerkinElmer, Waltham, MA, USA) under nitrogen gas flow. The blends were heated at 200 °C for 3 min to erase the thermal history and fast quenched to 0 °C before scanning from 0 to 200 °C at a heating rate of 10 °C/min. The *DSC-X_c,hc_* of the blends was also calculated from Δ*H_m,hc_* and enthalpy of cold crystallization (Δ*H_cc_*) using the Equation (3). After the DSC heating scans, the blends were kept at 200 °C for 3 min before being scanned from 200 to 0 °C at a cooling rate of 10 °C/min for DSC cooling scans.

DSC-*X_c,hc_* (%) = [(Δ*H_m,hc_* − Δ*H_cc_*)/(93.6 × *W_PL__LA3251D_*)] × 100 (3)

where *W_PLLA3251D_* is the weight fraction of PLLA3251D

For measurement of half crystallization-time (*t_1/2_*), the thermal history of blends was completely removed by heating at 200 °C for 3 min before quenching to 120 °C at a cooling rate of 50 °C/min. After that, the blends were isothermally scanned at 120 °C until the completion of crystallization [27]. The *t_1/2_* is the time required to obtain 50% of the final crystallinity.

Crystalline structures of the blend films were recorded using an XRD (D8 Advance, Bruker Corporation, Karlsruhe, Germany), as described above. The degree of crystallinity for PLLA homo-crystallites from XRD (XRD-*X_c,hc_*) of the blend films was calculated using Equation (4).

XRD-*X_c,hc_* (%) = (*S_c,hc_*/*S_a_*) × 100 (4)

where *S_c,hc_* and *S_a_* are the integrated intensity peaks for PLLA homo-crystallites and the integrated intensity of the amorphous halo, respectively.

## 3. Results

### 3.1. Characterization of LMW-PLLA and scPLA Powders

**Figure 1:**
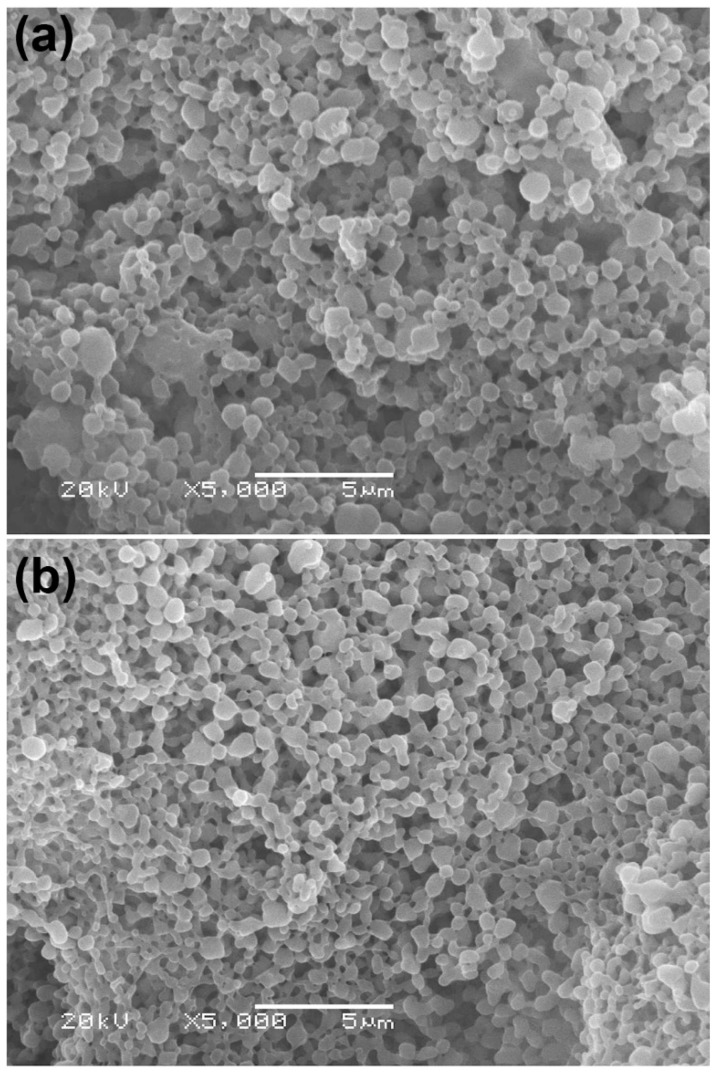
SEM images of (**a**) LMW-PLLA and (**b**) scPLA powders (All bar scales = 5 µm).

The particle morphology of LMW-PLLA and scPLA powders was investigated from the SEM images, as illustrated in Figure 1. Particles had nearly spherical shapes and smooth surfaces, indicating that the precipitation method in this work can prepare both the LMW-PLLA and scPLA powders with spherical shapes from the LMW-PLLA and LMW-PLLA/LMW-PDLA blend solutions, respectively. Most microspheres were less than 1 µm in diameter. Averaged particle sizes of the LMW-PLLA and scPLA powders were 610 ± 124 nm and 564 ± 108 nm, respectively, as determined by light-scattering analysis.

**Figure 2:**
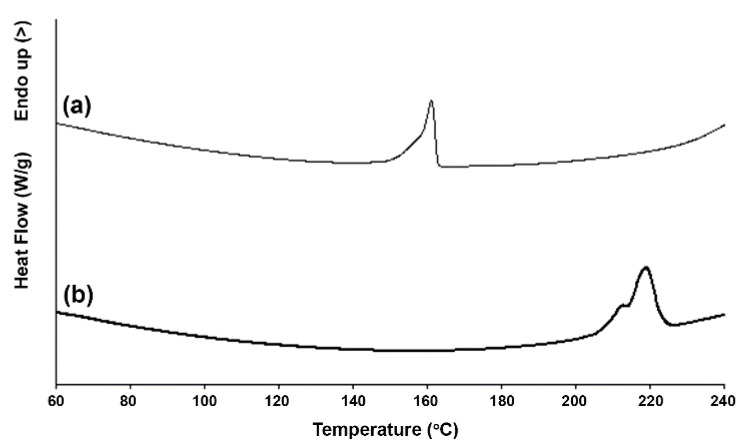
DSC heating curves of (**a**) LMW-PLLA and (**b**) scPLA powders.

Figure 2 shows the DSC heating curves of LMW-PLLA and scPLA powders, and the DSC results are summarized in Table 2. The LMW-PLLA and scPLA powders exhibited single *T_m_* peaks at 161 °C of homo-crystallites and at 219 °C of stereocomplex crystallites, respectively. This indicates that the scPLA powder was a complete stereocomplex formation [11,12]. The DSC-*X_c,hc_* and DSC-*X_c,sc_* values were 54.2 and 59.9 J/g for the LMW-PLLA and scPLA powders, respectively. The obtained powders of LMW-PLLA (610 ± 124 nm) and scPLA (564 ± 108 nm) were slightly different in particle size. From the DSC results in Table 2, the LMW-PLLA powder was completely homo-crystalline, whereas the scPLA powder was completely stereocomplex crystalline. Therefore, the particle sizes of these powders did not influence their DSC-*X_c,hc_* and DSC-*X_c,sc_* values.

**Figure 3:**
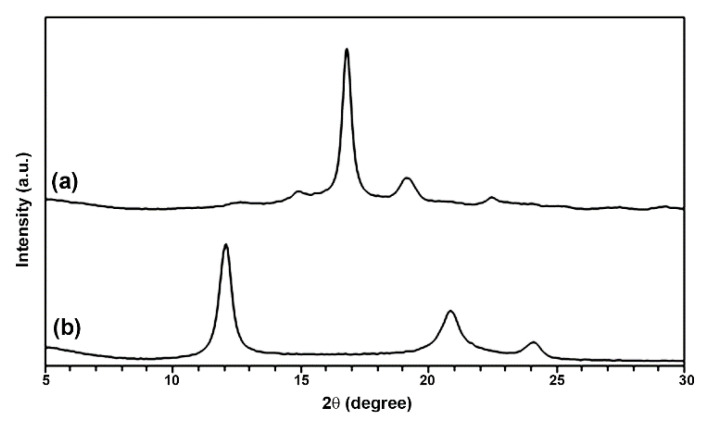
XRD patterns of (**a**) LMW-PLLA and (**b**) scPLA powders.

XRD patterns were used to investigate the crystalline structures of the powders, as presented in Figure 3. It can be seen that the LMW-PLLA powder exhibited diffraction peaks at 2θ = 14.8°, 16.9°, 19.1°, and 22.5° assigned to PLA homo-crystallites, whereas the scPLA powder showed diffraction peaks at 2θ = 12.0°, 20.8°, and 24.0° attributed to PLA stereocomplex-crystallites [17,28,29]. The XRD results confirmed that the LMW-PLLA and scPLA powders had complete homo- and stereocomplex crystallites, respectively, in agreement with the DSC results described above.

### 3.2. Characterization of PLLA3251D/LMW-PLLA Powder and PLLA3251D/scPLA Powder Blends

#### 3.2.1. Phase Morphology

**Figure 4:**
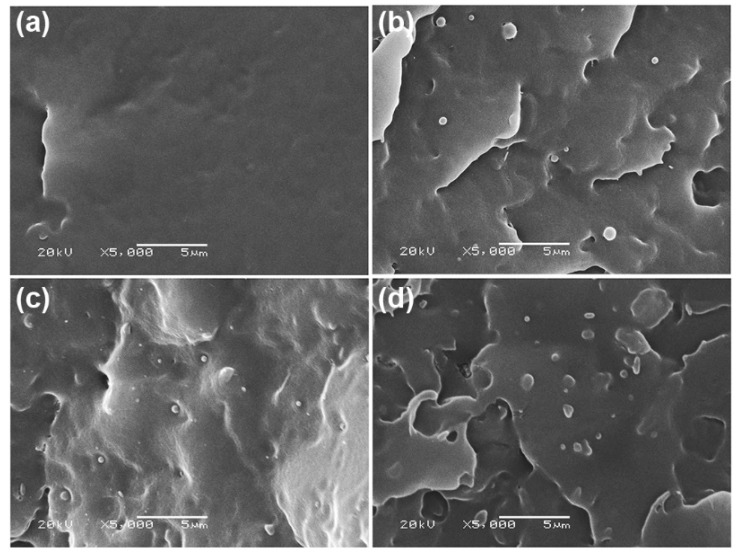
SEM images of cryogenically fractured surfaces of (**a**) pure PLLA3251D film and PLLA3251D/scPLA powder films with scPLA powder contents of (**b**) 2%, (**c**) 4%, and (**d**) 8% *w*/*w* (All bar scales = 5 µm).

The phase morphology of the blend films was determined from their SEM images of cryogenically fractured surfaces. The pure PLLA3251D film had no phase separation, as shown in Figure 4a. All the PLLA3251D/LMW-PLLA powder-blend films also exhibited homogeneous films similar to the pure PLLA3251D film, as shown in Figure S1. This was due to the LMW-PLLA powder having a low *T_m_* (161 °C) and being completely melted during blending at 180 °C. For the PLLA3251D/scPLA powder-blend films, it can be observed that the particles of scPLA were clearly detected on the PLLA3251D matrices, as shown in Figure 4b–d, indicating that the scPLA powder did not deform during melt blending and hot pressing at 180 °C. This is because the *T_m_* of the scPLA powder was 219 °C. In addition, the scPLA powder contents of 2%, 4%, and 8% w/w in this work were well-distributed and dispersed in the PLLA3251D matrices and the surfaces of the scPLA particles attached with the PLLA3251D matrices, implying that the PLLA3251D matrices and scPLA powder had good compatibility.

#### 3.2.2. Thermal Transition Properties

**Figure 5:**
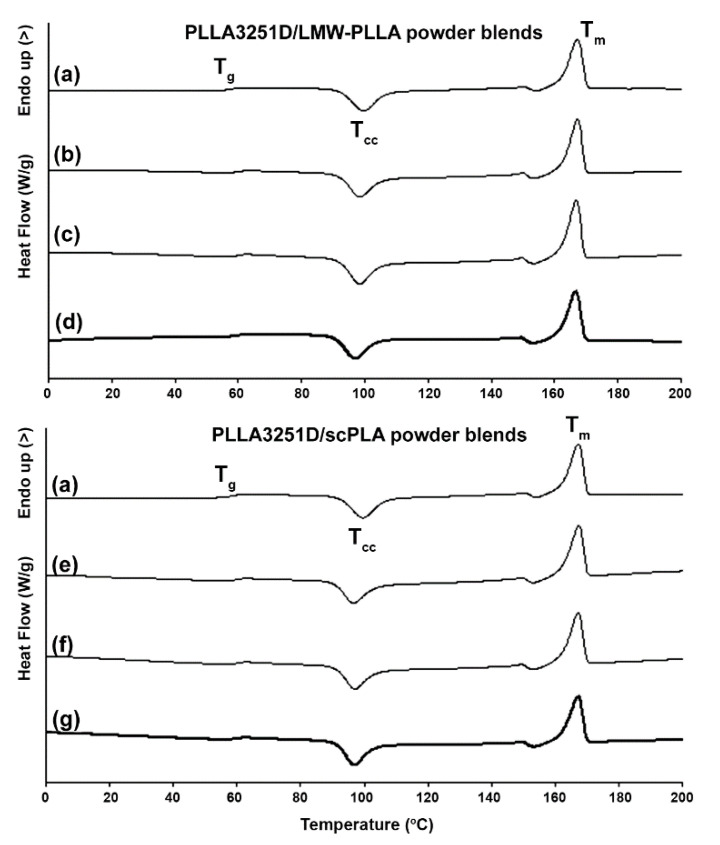
DSC heating curves of (**a**) pure PLLA3251D and PLLA3251D/LMW-PLLA powder blends with powder contents of (**b**) 2%, (**c**) 4%, and (**d**) 8% *w*/*w* as well as PLLA3251D/scPLA powder blends with powder contents of (**e**) 2%, (**f**) 4%, and (**g**) 8% *w*/*w* (*T_g_*, *T_cc_* and *T_m_* peaks as shown).

The crystallization properties of the blend samples were investigated using DSC methods. Figure 5 shows the DSC heating scans, and the DSC results are summarized in Table 3. The *T_g_* and *T_m_* values of both the PLLA3251D/powder blends were in the range 58–60 °C and 166–167 °C, respectively. The *T_cc_* peaks of the PLLA3251D/LMW-PLLA powder and PLLA3251D/scPLA powder blends slightly decreased as the powder content increased. This may be explained by a plasticization effect of the LMW-PLLA blending and a nucleation effect of the scPLA powder addition for the PLLA3251D/LMW-PLLA powder and PLLA3251D/scPLA powder blends, respectively. The LMW-PLLA powder was homogeneously melt-blended with the PLLA3251D matrix. The LMW-PLLA fractions enhanced the chain mobility of PLLA3251D to induce the plasticization of PLLA3251D [30,31], while the scPLA powder acted as a heterogeneous nucleating agent to accelerate the crystallization of PLLA3251D [31,32].

The DSC-*X*_c,hc_ values of PLLA3251D (8.6%) did not change significantly by blending with the LMW-PLLA powder (8.7–9.8%), as reported in Table 3. However, the DSC-*X_c,hc_* values of the PLLA3251D/scPLA powder blends steadily increased as the scPLA-powder content increased. This confirms that the scPLA powder acted as a nucleating agent for PLLA3251D.

**Figure 6:**
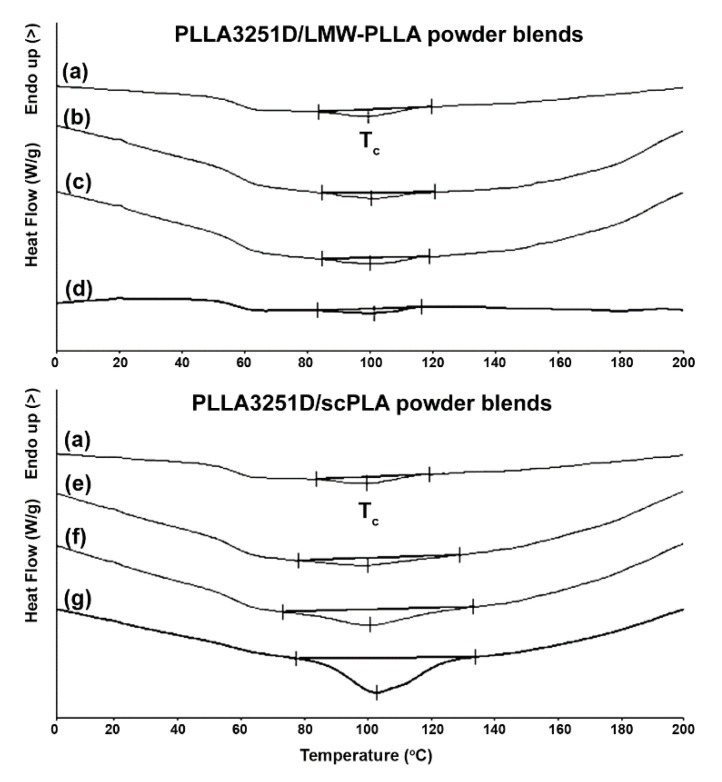
DSC cooling curves of (**a**) pure PLLA3251D and PLLA3251D/LMW-PLLA powder blends with powder contents of (**b**) 2%, (**c**) 4%, and (**d**) 8% *w*/*w* as well as PLLA3251D/scPLA powder blends with powder contents of (**e**) 2%, (**f**) 4%, and (**g**) 8% *w*/*w* (*T_c_* peaks as shown).

The crystallization properties of the blends were also determined from DSC cooling scans, as shown in Figure 6, and are summarized in Table 4. *T_c_* peaks and Δ*H_c_* values of PLLA3251D/LMW-PLLA powder blends did not significantly change as the LMW-PLLA powder content increased, suggesting that the blending of the LMW-PLLA powder did not affect the crystallization of the PLLA3251D matrices upon DSC cooling scans. For the PLLA3251D/scPLA powder blends, the *T_c_* peaks were slightly shifted to higher temperatures and Δ*H_c_* values greatly increased as the scPLA powder content increased, indicating that the scPLA powder enhanced the crystallizability of PLLA3251D [18,31] according to the DSC-*X_c,hc_* results from the DSC heating scans.

**Figure 7:**
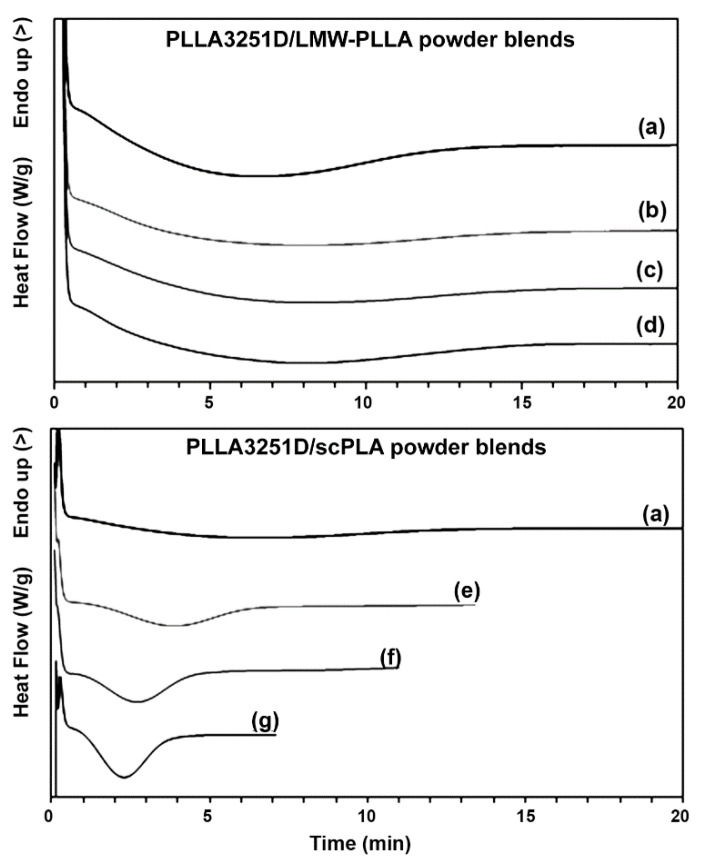
DSC isothermal curves at isothermal temperature of 120 °C of (**a**) pure PLLA3251D and PLLA3251D/LMW-PLLA powder blends with powder contents of (**b**) 2%, (**c**) 4%, and (**d**) 8% *w*/*w* as well as PLLA3251D/scPLA powder blends with powder contents of (**e**) 2%, (**f**) 4%, and (**g**) 8% *w*/*w*.

**Figure 8:**
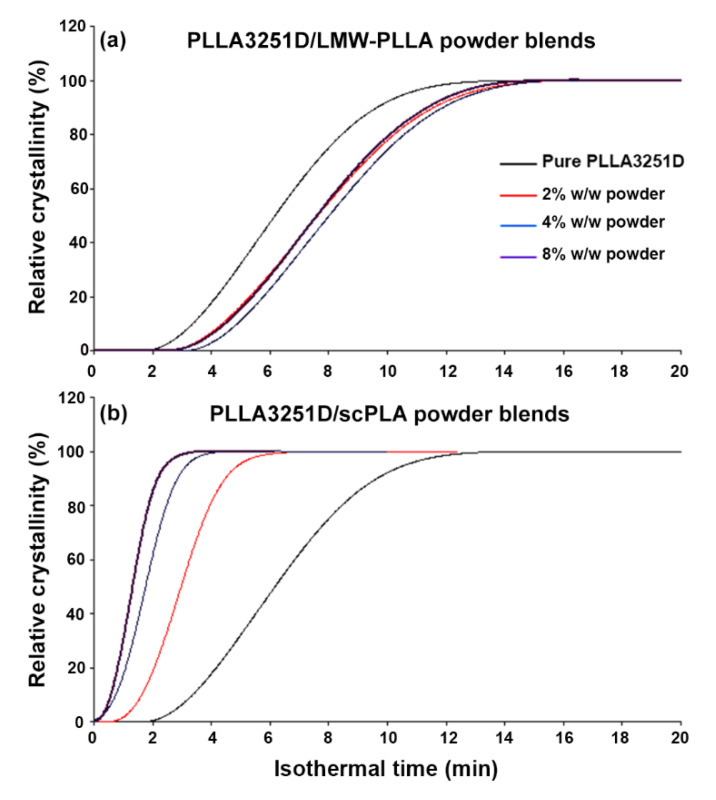
Relative crystallinity as a function of time of (**a**) PLLA3251D/LMW-PLLA powder and (**b**) PLLA3251D/scPLA powder blends.

The half-crystallization time (*t_1/2_*) of the polymers has usually been used to investigate the effectiveness of nucleating agents [8,27]. DSC isothermal curves and relative crystallinity as a function of time of the blend samples are presented in Figure 7 and Figure 8, respectively. It was found that the isothermal crystallization peaks of the PLLA3251D matrices in Figure 7 became sharper with the addition of the scPLA powder (Figure 7e–g) but the LMW-PLLA powder did not have that effect (Figure 7b–d). These sharper peaks were assigned to a shorter time for crystallization [33]. The *t_1/2_* values obtained from Figure 8 are reported in Table 5. The pure PLLA3251D had a *t_1/2_* of 6.17 min. The *t_1/2_* values of the PLLA3251D/LMW-PLLA powder blends were in the range of 7.59–8.02 min, similar to pure PLLA3251D (6.17 min). Meanwhile, the *t_1/2_* values of the PLLA3251D/scPLA powder blends dramatically decreased from 6.17 min to 2.96 min when the 2% w/w scPLA powder was added. With increasing scPLA powder content, the *t_1/2_* of the blends steadily decreased. The *t_1/2_* results confirmed that the scPLA powder acted as an effective nucleating agent to increase the crystallization rate of the PLLA3251D matrices [34,35], whereas the LMW-PLLA powder did not affect the crystallization rate.

#### 3.2.3. Crystalline Structures

**Figure 9:**
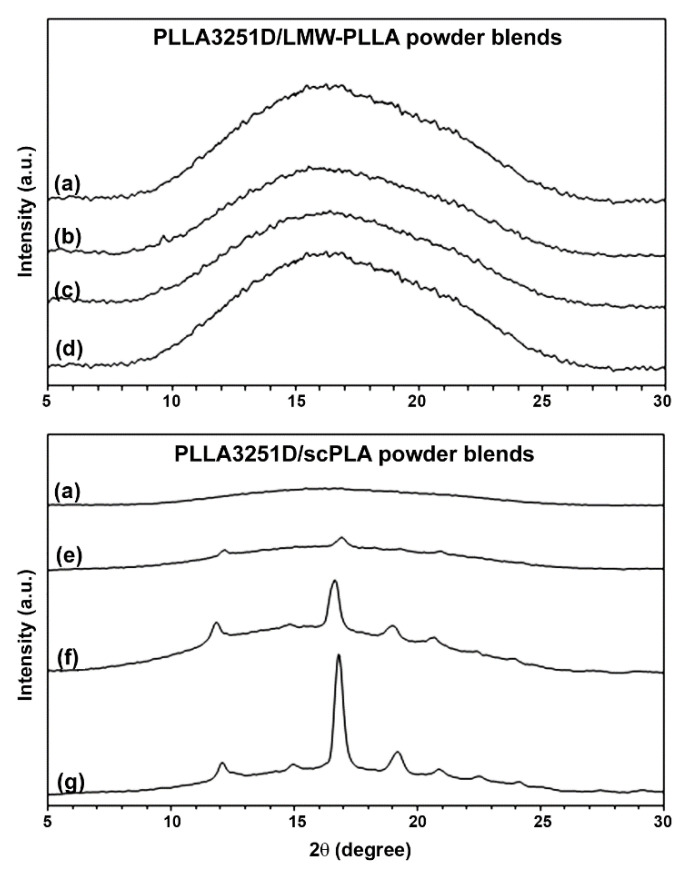
XRD patterns of (**a**) pure PLLA3251D and PLLA3251D/LMW-PLLA powder blend films with powder contents of (**b**) 2%, (**c**) 4%, and (**d**) 8% *w*/*w* as well as PLLA3251D/scPLA powder blend films with powder contents of (**e**) 2%, (**f**) 4%, and (**g**) 8% *w*/*w*.

The crystalline structures of the blend films were determined from the XRD patterns, as presented in Figure 9. The pure PLLA3251D film had no diffraction peaks (Figure 9a), implying that it was completely amorphous. This is due to the HMW-PLLA only slowly crystallizing after processing [8,16]. All the PLLA3251D/LMW-PLLA powder films (Figure 9b–d) were also completely amorphous. The LMW-PLLA powder was homogeneously melted with the PLLA3251D matrix, as observed from the above SEM results. This suggests that the LMW-PLLA blending could not enhance the crystallization of the PLLA3251D matrix according to the DSC results.

The PLLA3251D/scPLA powder films showed diffraction peaks of stereocomplex crystallites for dispersed scPLA powder at 2θ = 12.0°, 20.8°, and 24.0° [17,28,29] (Figure 9e–g). The scPLA powder with complete stereocomplexation (see Figure 3) was well-dispersed in the PLLA3251D matrices (see Figure 4). It can be seen that the diffraction peaks of the PLA homo-crystallites appeared at 2θ = 14.8°, 16.9°, 19.1°, and 22.5° [17,28,29] when the scPLA powder was blended. The intensities of the diffraction peaks for PLA homo-crystallites significantly increased as the scPLA powder content increased. These diffraction peaks attributed to the α phase of the PLA homo-crystallites [36]. It should be noted that the diffraction peaks with weak intensities at 2θ = 14.8° and 22.5° were observed for PLLA3251D/scPLA powder films, suggesting mixtures of α and α′ crystalline phases [36,37]. The XRD-*X_c,hc_* values of these blend films calculated from Equation (4) were 2.57%, 8.05%, and 22.57% for the 2%, 4%, and 8% w/w scPLA powder contents, respectively. The XRD results supported a conclusion that the scPLA powder acted as an effective nucleating agent for PLLA3251D, which is in agreements with the DSC results.

## 4. Conclusions

LMW-PLLA and scPLA powders with nearly spherical shapes and smooth surfaces were successfully prepared using the precipitation of LMW-PLLA and LMW-PLLA/LMW-PDLA solutions, respectively. The resulting LMW-PLLA and scPLA powders had single *T_m_* peaks at 161 °C and 219 °C, respectively, as determined by DSC, attributed to complete homo- and sterecomplex crystallites, respectively, as revealed by XRD. The nucleation effectiveness of the scPLA powder was investigated by melt blending with PLLA3251D compared to the LMW-PLLA powder. The scPLA powder showed good compatibility and was well-dispersed with the PLLA3251D matrices, whereas the LMW-PLLA powder was homogeneously melted in the PLLA3251D matrices, as observed by SEM. From DSC and XRD analyses, the crystallinity content of the PLLA3251D-based blends significantly increased as the scPLA powder content increased, but the LMW-PLLA powder did not exhibit the same behavior. Therefore, it is possible to conclude that the scPLA powder prepared by the simple precipitation method can act as an efficient nucleating agent for HMW-PLLA. Fully biodegradable HMW-PLLA/scPLA powder biocomposites are very promising for biomedical, tissue engineering, and packaging applications.

## Figures and Tables

**Table 1:** *L*-enantiomer contents, molecular weight characteristics, and thermal properties of LMW-PLLA and LMW-PDLA.

LMW-PLA	*L*-content (%) ^a^	Theoretical *M.W.* (g/mol) ^b^	*M_n_* (g/mol) ^c^	*Ɖ* ^d^	*T_g_* (°C) ^e^	*T_m_* (°C) ^f^
LMW-PLLA LMW-PDLA	97.5 2.8	5000 5000	6700 6200	1.4 1.8	46 46	161 161

^a^*L*-enantiomer content determined from polarimetry. ^b^ theoretical molecular weight (*M.W.*) calculated from lactide/1-dodecanol ratio. ^c^ number-average molecular weight determined from gel permeation chromatography (GPC). ^d^ dispersity index determined from gel permeation chromatography (GPC). ^e^ glass transition temperature obtained from differential scanning calorimetry (DSC). ^f^ melting temperature obtained from differential scanning calorimetry (DSC).

**Table 2:** DSC results of LMW-PLLA and scPLA powders obtained from Figure 2.

Powder	*T_m_* (°C) ^a^	Δ*H_m,hc_* (J/g) ^b^	Δ*H_m,sc_* (J/g) ^c^	DSC-*X_c,hc_* (%) ^d^	DSC-*X_c,sc_* (%) ^e^
LMW-PLLA powder scPLA powder	161 219	50.8 -	- 85.1	54.2 -	- 59.9

^a^ melting temperature. ^b^ enthalpy of melting for homo-crystallites. ^c^ enthalpy of melting for stereocomplex crystallites. ^d^ degree of homo-crystallinity of powder from DSC calculated from Equation (1). ^e^ degree of stereocomplex crystallinity of powder from DSC calculated from Equation (2).

**Table 3:** Thermal transition properties of PLLA3251D-based blends obtained from DSC heating curves in Figure 5.

PLLA3251D-Based Blends	*T_g_* (°C) ^a^	*T_cc_* (°C) ^b^	Δ*H_cc_* (J/g) ^c^	*T_m_* (°C) ^d^	Δ*H_m_* (J/g) ^e^	DSC-*X_c,hc_* (%) ^f^
Pure PLLA3251D	58	110	32.5	167	40.6	8.6
PLLA/2% w/w LMW-PLLA powder	60	98	32.4	167	40.4	8.7
PLLA/4% w/w LMW-PLLA powder	60	98	33.4	167	42.2	9.8
PLLA/8% w/w LMW-PLLA powder	60	97	32	166	39.9	9.2
PLLA/2% w/w scPLA powder	60	97	27.8	167	40.4	14
PLLA/4% w/w scPLA powder	60	97	27	167	40.4	15.5
PLLA/8% w/w scPLA powder	59	96	24.8	167	40	19.1

^a^ glass-transition temperature. ^b^ cold-crystallization temperature. ^c^ enthalpy of cold crystallization. ^d^ melting temperature. ^e^ enthalpy of melting. ^f^ degree of homo-crystallinity of blends from DSC calculated from Equation (3).

**Table 4:** Crystallization properties of PLLA3251D/LMW-PLLA powder and PLLA3251D/scPLA powder blends from DSC cooling curves in Figure 6.

PLLA3251D-Based Blends	*T_c_* (°C) ^a^	Δ*H_c_* (J/g) ^b^
Pure PLLA3251D	100	3.8
PLLA/2% w/w LMW-PLLA powder	100	3.4
PLLA/4% w/w LMW-PLLA powder	100	3.8
PLLA/8% w/w LMW-PLLA powder	101	3.5
PLLA/2% w/w scPLA powder	100	6.2
PLLA/4% w/w scPLA powder	101	14.6
PLLA/8% w/w scPLA powder	103	28.2

^a^ crystallization temperature obtained from DSC cooling curves in Figure 6. ^b^ enthalpy of crystallization obtained from DSC cooling curves in Figure 6.

**Table 5:** Half-crystallization times (*t_1/2_*) of PLLA3251D/LMW-PLLA powder and PLLA3251D/scPLA powder blends from relative crystallinity as a function of time in Figure 8.

PLLA3251D-Based Blends	*t_1/2_* (min) ^a^
Pure PLLA3251D	6.17
PLLA/2% w/w LMW-PLLA powder	7.6
PLLA/4% w/w LMW-PLLA powder	8.02
PLLA/8% w/w LMW-PLLA powder	7.59
PLLA/2% w/w scPLA powder	2.96
PLLA/4% w/w scPLA powder	1.79
PLLA/8% w/w scPLA powder	1.32

^a^ half-crystallization time determined at 50% relative crystallinity in Figure 8.

## Data Availability

The data presented in this study are available on request from the corresponding author.

## Supplementary Materials

The following supporting information can be downloaded at: https://www.mdpi.com/article/10.3390/polym14194092/s1, Figure S1: SEM images of cryogenically fractured surfaces of (a) pure PLLA3251D film and PLLA3251D/LMW-PLLA powder films with LMW-PLLA powder contents of (b) 2%, (c) 4%, and (d) 8% w/w (All bar scales = 5 µm).
