# Supplementary material for: Enhancement in Crystallizability of Poly(L-Lactide) Using Stereocomplex-Polylactide Powder as a Nucleating Agent
Source: Polymers (Basel). 2022 Sep 29;14(19):4092. doi: 10.3390/polym14194092 (PMC9571414; doi:10.3390/polym14194092)
Supplement: Supplementary file 1 [file polymers-14-04092-s001.zip › polymers-1911010-supplementary.pdf]

Supplementary Materials

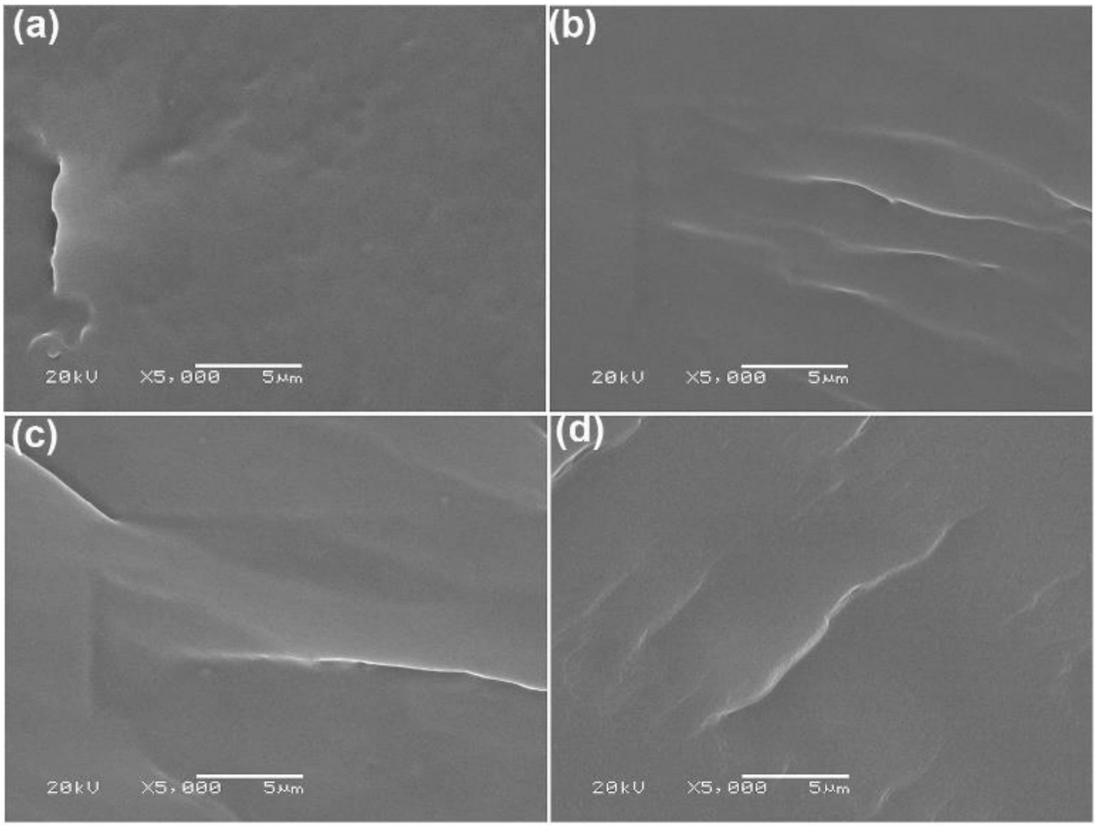

Figure S1. SEM images of cryogenically fractured surfaces of (**a**) pure PLLA3251D film and PLLA3251D/LMW-PLLA powder films with LMW-PLLA powder contents of (**b**) 2%, (**c**) 4%, and (**d**) 8% w/w (All bar scales = 5 µm).
